# Genetic variability of *Taenia solium* cysticerci recovered from experimentally infected pigs and from naturally infected pigs using microsatellite markers

**DOI:** 10.1371/journal.pntd.0006087

**Published:** 2017-12-28

**Authors:** Mónica J. Pajuelo, María Eguiluz, Elisa Roncal, Stefany Quiñones-García, Steven J. Clipman, Juan Calcina, Cesar M. Gavidia, Patricia Sheen, Hector H. Garcia, Robert H. Gilman, Armando E. Gonzalez, Mirko Zimic, for the Cysticercosis Working Group in Peru

**Affiliations:** 1 Laboratorio de Bioinformática y Biología Molecular, Facultad de Ciencias y Filosofía, Universidad Peruana Cayetano Heredia, Lima, Peru; 2 Department of International Health, Johns Hopkins Bloomberg School of Public Health, Baltimore, United States of America; 3 School of Veterinary Medicine, Universidad Nacional Mayor de San Marcos, Lima, Peru; 4 Center for Global Health, Universidad, Peruana Cayetano Heredia, Lima, Peru; 5 Department of Microbiology, School of Science and Philosophy, Universidad Peruana Cayetano Heredia, Lima, Peru; Ludwig-Maximilians-University, UNITED STATES

## Abstract

The adult *Taenia solium*, the pork tapeworm, usually lives as a single worm in the small intestine of humans, its only known definitive host. Mechanisms of genetic variation in *T*. *solium* are poorly understood. Using three microsatellite markers previously reported [1], this study explored the genetic variability of *T*. *solium* from cysts recovered from experimentally infected pigs. It then explored the genetic epidemiology and transmission in naturally infected pigs and adult tapeworms recovered from human carriers from an endemic rural community in Peru. In an initial study on experimental infection, two groups of three piglets were each infected with proglottids from one of two genetically different tapeworms for each of the microsatellites. After 7 weeks, pigs were slaughtered and necropsy performed. Thirty-six (92.3%) out of 39 cysts originated from one tapeworm, and 27 (100%) out of 27 cysts from the other had exactly the same genotype as the parental tapeworm. This suggests that the microsatellite markers may be a useful tool for studying the transmission of *T*. *solium*. In the second study, we analyzed the genetic variation of *T*. *solium* in cysts recovered from eight naturally infected pigs, and from adult tapeworms recovered from four human carriers; they showed genetic variability. Four pigs had cysts with only one genotype, and four pigs had cysts with two different genotypes, suggesting that multiple infections of genetically distinct parental tapeworms are possible. Six pigs harbored cysts with a genotype corresponding to one of the identified tapeworms from the human carriers. In the dendrogram, cysts appeared to cluster within the corresponding pigs as well as with the geographical origin, but this association was not statistically significant. We conclude that genotyping of microsatellite size polymorphisms is a potentially important tool to trace the spread of infection and pinpoint sources of infection as pigs spread cysts with a shared parental genotype.

## Author summary

*Taenia solium*, the pork tapeworm, is a major cause of epilepsy in developing countries. Although it has been deemed a potentially eradicable pathogen, it remains prevalent in rural communities. This two-part study aims to evaluate the utility of three microsatellite markers previously reported, to identify parasites and to establish relationships among them. In the first study, we evaluated the genetic variability of the progeny of two individual tapeworms by infecting two groups of three pigs each. We found variation of 8% and 0% in the two groups with respect to the parental tapeworm, indicating that the cysts source may be identifiable. Next, in the second study we described the genetic relationships among tapeworms obtained from four carriers and cysts obtained from eight naturally infected pigs in a rural community. We demonstrated that pigs can have two types of cysts, suggesting multiple infections. In addition, we found relatedness between 6 pigs and one tapeworm identified in the community. Our results indicate the potential for microsatellite markers to identify genetic relationships between parasites and thereby establish routes of transmission. It is likely that the limited number of microsatellites prevented us from establishing relatedness with more precision. Therefore, further evaluation of additional microsatellites is recommended.

## Introduction

*Taenia solium* is a zoonotic parasite that affects both humans and pigs. Larval infection in the human brain results in neurocysticercosis, the sole main cause of acquired adult epilepsy in developing countries [2]. Neurocysticercosis is considered a potentially eradicable disease [3], and many efforts to implement interventions to control or eliminate this parasite are currently being explored [4, 5]; however, there are some aspects of the epidemiology, such as distribution and transmission of the parasite among endemic communities that are still unknown. Control strategies for cysticercosis vary in scale and scope. Understanding the interaction between the parasite, swine host, and humans by observing transmission dynamics and clustering of infection through the use of genotyping can help determine the most cost-effective intervention for the given setting.

Although the genetic variation of *T*. *solium* has not yet been fully described, a detailed understanding of *T*. *solium* population genetic structure is vital to determining the transmission and other epidemiological features of this disease [6–8]. Moreover, the study of genetic variants may help elucidate the reproduction aspects of the parasite [8].

Genotyping tools are useful to study the etiology and distribution of parasites among populations [9, 10]. The genetic variability of *T*. *solium*, in particular, demonstrates geographic distribution that has been previously described in the literature [11–13]. In experimental infection, genetic variability has been shown among cysts originating from a given parental tapeworm, as well as an association between the genotypes of the parental tapeworm and its cysts progeny [14].

Given that *T*. *solium* is a monoecious self-fertilizing parasite, most common genetic markers show poor genetic variability, and therefore genetic markers with higher mutations rates, such as microsatellites are needed. Microsatellites have been used in other cestodes, like *Echinococcus multilocularis*, in which the, spatial distribution of strains was studied in different regions of the world [9]. In particular, this was analyzed at a local scale, in the French Ardennes area, Italy, Hungary, and Norway [15–18]. Remarkably, it was found that the use of the single microsatellite marker, EmsB, is sufficient for molecular tracking of transmission [19, 20]. Albeit not from public health perspective, the use of microsatellites has also been reported in the study of genetic and reproductive aspects of the cestode *Schistocephalus solidus* [21], as well as, in the Mendelian inheritance in *Oochoristica javaensis*[22].

Recently, our group has sequenced the whole genome of two *T*. *solium* isolates. After identifying more than 9,000 microsatellite sequences along the complete genome, we evaluated 36 microsatellites, and identified a set of seven polymorphic microsatellite markers with potential use for genotyping [1]. Using those markers, strains from communities in the North and South of Peru were able to be discriminated, and a small amount of intra-community genotypic variability was able to be detected [1].

This two-pronged investigation including one experimental and one epidemiologic study was developed to evaluate *T*. *solium* genetic variability in a public health context. The experimental portion of this study aimed to establish empirical evidence that offspring cysts have the same genotype as the parental tapeworm. We evaluated the genotypes of offspring cysts derived from pigs experimentally infected with *T*. *solium* proglottids of the same tapeworm. Experimental evidence was designed to inform the second part of this study, and is important in the ability to pinpoint sources of infection and allow for epidemiologic tracing. The epidemiologic study aimed to examine associations between tapeworms and cysts found in pigs. We analyzed the association of genotypes of tapeworms from human carriers and cysts from naturally infected pigs in a rural community in Piura, a northern city of Peru to provide an initial exploratory assessment of the epidemiology of transmission in this community.

## Materials and methods

### Genetic variation in cysts developing in pigs infected with eggs from the same tapeworm

The objective of this experiment was to identify and evaluate the spontaneous variability of cyst genotypes compared to the genotypes of the parental tapeworm from which they originated. For this, two groups of three pigs each were experimentally infected with proglottids of two different tapeworms with distinct known genotypes based on four microsatellite markers.

#### Tapeworm samples

The *T*. *solium* samples were donated from the repository of the Cysticercosis Working Group in Peru, and they were obtained from “complete” tapeworms (e.g. portion very close to the scolex and gravid proglottids) expelled as residual samples after routine treatment of two patients, one from Apurímac in the South Highlands of Peru (Tapeworm TA) and one from Cajamarca in the North Highlands of Peru (Tapeworm TB). Gravid proglottids were stored in 25% glycerol supplemented with penicillin (1000 UI/mL), gentamicin (100 μg/mL), amphotericin B (0.02 mg/mL), and streptomycin (1 mg/mL) at 4°C until infection. The proximal portion next to the scolex was stored in absolute ethanol at room temperature until genotyping was performed. Both had different genotypes, evaluated by sequencing (described below in the Genotyping section). The closest segment to the scolex portion of the parasite was used for DNA extraction and genotyping the tapeworm, since it does not have proglottids with eggs [23]. The gravid proglottids, which proved to be viable (see below), were used to experimentally infect pigs.

#### Animals

Six one-month-old female piglets (*Sus scrofa domestica*) were obtained from a farm free of *T*. *solium* in Lima, Peru. Animals were confirmed to be negative to the antibody detection assay by an enzyme-linked immunoelectrotransfer blot (EITB, western blot) assay using lentil-lectin purified parasite glycoprotein antigens [24].

#### Viability of oncospheres

To proceed to the artificial infection of pigs, it was important to know if the oncospheres contained in the proglottids were viable. This was done as described by Verástegui et al [25]. Briefly, the oncospheres were released using hypochlorite at 0.75% for 10 minutes to weaken the egg layer. Subsequently they were washed 3 times with RPMI 1640 medium, 0.4% trypan blue added. It was observed under the microscope and the oncospheres that excludes the dye were considered viable.

#### Infection of pigs

Three piglets were infected with one proglottid from tapeworm TA each, and the other three with proglottids from tapeworm TB. Infection was carried out seven months after tapeworms were obtained.

Pigs were identified with a numbered ear-tag. Proglottids were administered inside a piece of banana as previously described [26]. Infection was confirmed by a positive EITB Western blot test [24] two weeks after infection.

#### Porcine blood sampling

Blood was obtained from the animals for cysticercosis serology two weeks after infection. A trained veterinarian collected the blood sample from the anterior cava vein using vacuum tubes; serum was isolated by centrifugation at 3500 r.p.m. for 5 minutes. Samples were stored at -20°C.

#### Necropsy

All pigs were euthanized 7 weeks post infection. Each pig was injected with Ketamine (20 mg/kg) and Xylacine (2 mg/kg) IM to produce sedation. Under sedation, the pigs were injected with sodium pentobarbital IV to produce euthanasia. Necropsy was performed immediately after euthanasia [26]. Full carcass dissections were performed. Healthy cysts were recovered from each carcass.

#### Collection of cysts for DNA extraction

Healthy cysts were individually and randomly selected and collected from the trunk, muscles from legs and arms, brain and heart, if available for each pig. Cysts were washed with saline solution 0.9% twice and a final wash with ethanol, so the sample is free from porcine tissue. Then each cyst was stored in absolute ethanol at 4°C. **A healthy cyst was defined** as a sack containing a transparent clear fluid and a white structure called the scolex. Cysts in this form are known to be mostly viable [27].

#### Cyst DNA purification

DNA purification was done using the QIAmp DNA Mini Kit (QIAGEN, Hilden, Germany) according to manufacturer´s instructions. The DNA quality and quantity was verified by UV spectrometry (Nanodrop 2000c) [28] and was stored at -20°C until use.

#### Microsatellite genotyping

Four microsatellite markers were used in this study: TS_SSR09, TS_SSR27, TS_SSR28, and TS_SSR32. Other markers reported in our previous study were excluded due to the following reasons: TS_SSR01 had shown low polymorphism among northern strains [1]. TS_SSR16 and TS_SSR18 were dinucleotides, with a high risk of unprecise assessment of polymorphism. Sanger sequencing was performed to assess size polymorphisms of the microsatellites. This technique causes loss of information at the 5´ end, which could include the repetitive motif of the microsatellite, therefore we designed a new set of external primers for the specific microsatellites SSR09, SSR27, SSR28, and SSR32 respectively: TS_SSR09-F (5´- TGGCATTCGACTGGATGACC -3´), TS_SSR09-R (5´- AGAGAAGCAACAGAATACTGC -3´), TS_SSR27-F (5´- AGGTAGACCACCTCCGTCTC -3´), TS_SSR27-R (5´- GGAAATTCGCATGGCTGTGG -3´), TS_SSR28-F (5´- TCTACCCCGTCAGTTGAGGT -3´), TS_SSR28-R (5´- GGTGTGAATTAACCAGCTAG -3´), TS_SSR32-F (5´- GGATGTGACGGGGTTTGACA -3´), and TS_SSR32-R (5´- CATTAGGGGTTCAGTCGGGG -3´) using Primer3 [29, 30]. The PCR reaction volume (25 μL) consisted of: Buffer 1X (Invitrogen), 2 mM MgCl_2_, 0.2 mM dNTPs each one, Forward primer: 1μM, Reverse primer: 1 μM and 20 ng of DNA, Taq polymerase 0.3 U. PCR was conducted in a MJ Research MiniCycler PTC-150 thermocycler with a hot cover using the following temperature profile except where otherwise stated: the initial denaturation step was at 95°C for 5 minutes, followed by 35 cycles of 95°C for 45 seconds, 62°C for 45 seconds and 72°C for 2 minutes and a final extension at 72°C for 5 minutes. PCR products were sent for DNA sequencing at Macrogen Corp., Rockville MD. The size of the microsatellite markers was calculated using a reference sequence obtained in our previous study, for comparison [1]. We verified that the variation in size was due to the number of repeats and not due to any other mutation in the flanking regions of the microsatellite marker.

### Analysis of cysts found in naturally infected pigs

The aim of this experiment was to evaluate the genetic variability of *T*. *solium* cysts from pigs recovered in a natural environment and its association with tapeworm carriers.

#### Design

First, a mapping and census of all houses was done in the community including GPS locations of each house. Tapeworm carriers had been previously identified three months prior in a previous study [31] and tapeworms were obtained from that study. Infected pigs were detected by tongue examination [32]. These animals were euthanized, and cysts were randomly recovered from the entire carcasses. Tapeworms and cysts were genotyped using the four microsatellites markers described above [1].

#### Study site

Pampa Elera Baja is a rural community located in the highlands of the Northern Region of Piura, in Peru. It has about 700 inhabitants. The prevalence of *T*. *solium* taeniosis was estimated in 1% [31]. 79/170 (42%) of families raised pigs at small scale.

#### Mapping and census

Mapping and a census of all houses in the community was performed. Mapping included geographic location (latitude and longitude coordinates) of each house recorded using global positioning system (GPS) receivers (GeoExplorer CE XT; Trimble, Westminster, CO). For the census, we obtained additional information from each person residing in the house, including age, sex, origin and characteristics of the house, such as material of construction, presence of latrine, disposal of feces, source of water, and treatment of water for consumption. GPS locations of houses that own pigs were used for analysis of association between genetic distances and geographic distances.

#### Tongue examination

Tongue examination was performed to identify possibly infected pigs [32]. Tongue examination is 100% specific [32]. The tongue of each pig was held using a special forceps while a trained veterinarian conducted a manual examination starting at the base of the tongue and palpating down to the tip to detect the presence of nodules and cysts. Pigs were considered positive if cysts were observed or palpated in the tongue muscle or base, otherwise the pig was considered negative.

#### Necropsy

Pigs were purchased from their owners and moved to a separate euthanasia area. Necropsy was performed as explained in the previous section. Healthy cysts were recovered from each carcass for microsatellite genotyping.

#### Microsatellite genotyping

DNA from tapeworms recovered from human carriers was previously obtained by our group (Watts, 2014) 3 months before, therefore no important variations at the population level are expected. DNA from cysts was collected from naturally infected pigs. All DNA samples collected were used for genotyping. All tapeworms and 10 to 14 cysts per pig were processed for DNA extraction, DNA purification, PCR amplification, and sequencing for microsatellites TS_SSR09, TS_SSR27, and TS_SSR28 as explained in previous sections.

#### Statistical genetic analysis

Expected heterozygosity was estimated with Arlequin V.3.5 software [33]. To determine the genetic distances between tapeworms and the cysts from different pigs, pairwise Nei’s genetic distance(Da) was calculated using POPULATIONS software version 1.2.32 [34]. A UPGMA (unweighted pair group method with arithmetic mean) dendrogram was inferred and trees were constructed using the FigTree software (http://tree.bio.ed.ac.uk/software/figtree/).

The geographic distances were calculated using data from the GPS records and the on-line distance calculator: Movable Type Scripts (http://www.movable-type.co.uk/scripts/latlong.html). The geographic location of the pigs was considered identical to the geographical location of the owner household. The associations between the genetic distances (Da) and geographic distances were tested by the Mantel test [35] using R [36].

The probability that a naturally infected pig had different types of cysts in proportions due to the natural variation was calculated by the binomial test.

### Ethics statement

All procedures complied and were **approved** by the Ethics Committee for Animal Use (CIEA: Assurance NumberA5146-01) at Universidad Peruana Cayetano Heredia (Lima, Peru) under Protocol Number 62400 for the experimental infection, and Protocol Number 61340 for the field study. *T*. *solium* tapeworms used for the experimental infections were donated from the repository of the Cysticercosis Working Group in Peru, and had been obtained from tapeworms expelled as residual samples after routine treatment. *T*. *solium* tapeworms used in the field study were obtained from a previous study conducted in the community of Pampa Elera [31].

## Results

### Genetic variation in cysts developing in pigs infected with eggs from the same tapeworm

Before infection, viability was established, Tapeworm TA had a viability of 68% and Tapeworm TB had a viability of 62%. All the pigs were successfully infected, as confirmed by necropsy. We processed a total of 39 healthy cysts from Tapeworm TA and 27 healthy cysts from Tapeworm TB. The two tapeworms showed different genotypes for all markers, except for SSR32 (Table 1).

**Table 1:** Genotypes of tapeworms used to experimentally infect pigs obtained by sequencing.

	SSR09 (GGT)	SSR27 (GAA)	SSR28 (GTA)	SSR32 (AGC)
Tapeworm TA	169	168	224	176
Tapeworm TB	160	153	221	176

Microsatellite SSR32 was found to be monomorphic among tapeworms TA and TB, and among cysts from different pigs (results are shown in S1 Table); therefore, it was not included in the analysis. Based on sequencing results, we defined that two genotypes were the same if the size bands in the three loci for SSR09, SSR27, and SSR28 were identical. Thirty six out of 39 examined cysts (92.3%, 95%CI: [79.1% - 98.4%]) from Tapeworm TA and 27 out of 27 examined cysts (100%, 95% CI: [87.2%-100%]) from Tapeworm TB showed the same genotype as the parental tapeworm (Table 2, and S1 Table). There are spontaneous mutations that appear to occur naturally and are not higher than two repeats (6 nucleotides). Therefore, the probability that a cyst with a difference of more than two repeats be a spontaneous evolution of the parental tapeworm is 7.7%. In analyzing each individual marker, 1/39 cysts varied in any of the markers (2.6%, 95% CI [0.1%-13.5%]). The results of the markers that differed from the parental tapeworm were amplified and sequenced twice and the differences were consistently observed.

**Table 2:** Genotype of cysts from experimental infection based on sequencing.

Pig	Number of cysts found at necropsy	Number of examined cysts	SSR09	SSR27	SSR28
A1	1245	10	169	168	224
		1	169	168	**218**^a^
		1	169	**165**^a^	224
A3	499	13	169	168	224
		1	**163**^a^	168	224
A7	1611	13	169	168	224
B4	675	11	160	153	221
B5	11	5	160	153	221
B6	135	11	160	153	221

Pigs A1, A3 and A7 were infected with Tapeworm TA proglottids and Pigs B4, B5 and B6 were infected with Tapeworm TB proglottids.

^a^ Alleles that were different (in size) from the original tapeworm allele

### Analysis of cysts found in naturally infected pigs

#### Census and mapping

**Fig 1:**
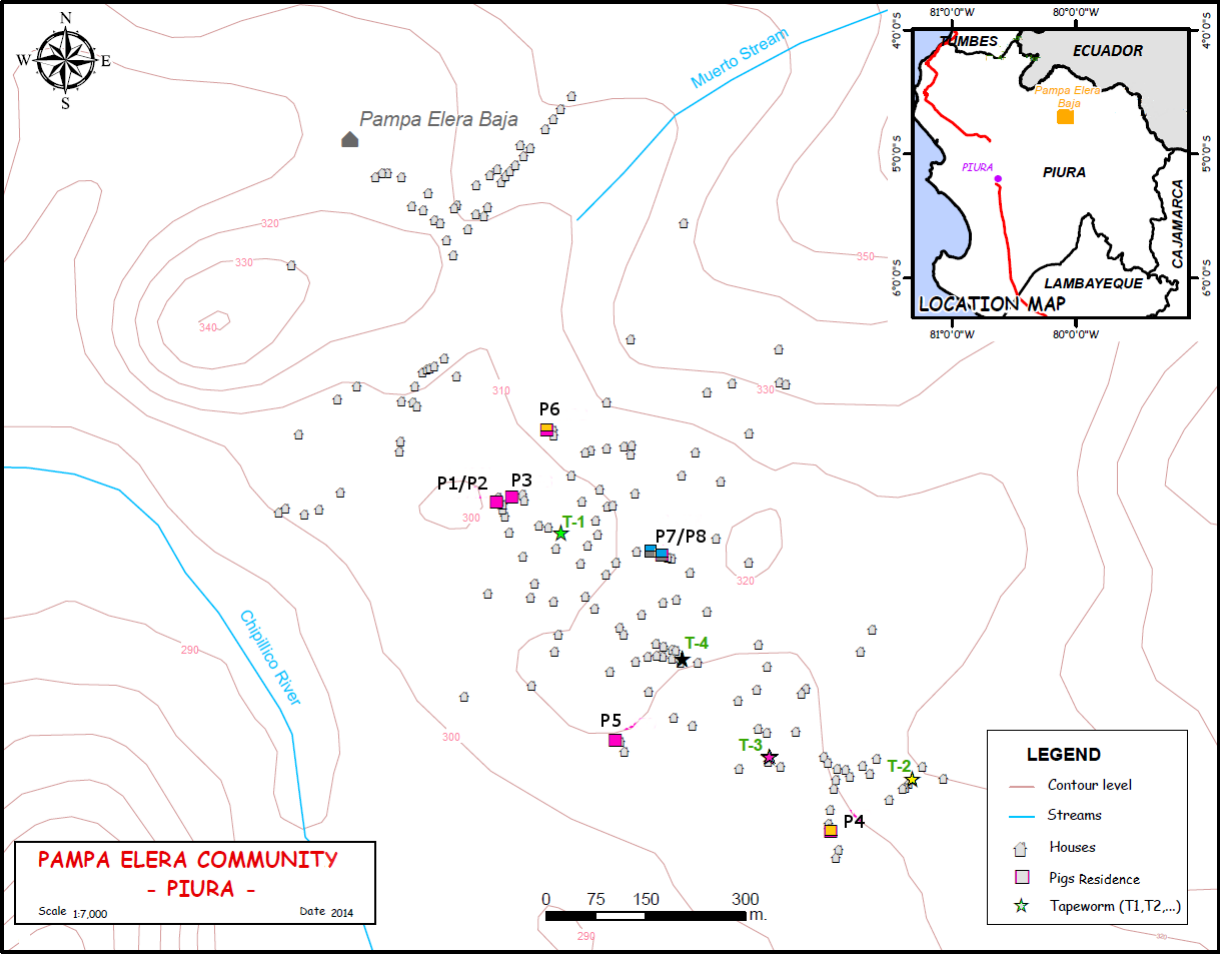
Map of the community showing the location of tapeworm carriers and cysticercosis-positive pigs. This figure was created using ArcGIS and http://escale.minedu.gob.pe/descargas/mapa.aspx was used also for base layer. Colors represent the genotype of tapeworms and cysts in pigs.

Pampa Elera Baja is a typical rural community with absence of sanitary facilities, where pigs roam freely to forage for food. A total of 530 people were surveyed in 170 houses in the community. Two hundred and seventy one (51.2%) were women and 259 (48.8%) were men. Median age of the population was 26 years old (IQR 11–45). This is a rural community where 90% of the houses were made of regional material (wood and mat). Ninety four percent of the population consumed water from river or ditch, 74% reported treatment of water for consumption, and 20% consumed untreated water. Eighty eight percent of the households did not have a latrine, and 6% had an artisanal latrine. Eighty six percent of the population reported to defecated in the field, while only 8% did in a latrine. Regarding porcine husbandry, a total of 79 families (46%) declared that they raised pigs. Families raised 3.5 pigs on average, ranging from 1 to 12 per family. The map of the village, showing the location of infected pigs and tapeworm carriers identified in a previous study [31] is shown in Fig 1.

Among the four tapeworm carriers, two of them (3 and 4) did not have a latrine and declared that they defecated in the field. The other two tapeworm carriers (1 and 2) declared that they had a latrine and used them.

#### Identification of infected pigs

During field work, a total of 303 pigs were evaluated, more than what was declared during census. A total of 9 tongue positive pigs were identified, of which 8 were slaughtered and healthy cysts were collected per animal. All infected pigs were born and raised in Pampa Elera Baja, they all roamed freely as stated by the owner. The characteristics of the animals are described in Table 3.

**Table 3:** Characteristics of pigs with cysticercosis used in this study.

Pig	Sex	Age (months)	House	Number of cysts found (counted)	Number of cysts evaluated
P1	Male	7	117	2276	15
P2	Male	7	118	959	13
P3	Male	7	13	1837	12
P4	Female	9	79	634	10
P5	Male	9	91	1448	10
P6	Female	12	1	65	10
P7	Female	24	40	1064	12
P8	Female	24	40	114	14

#### Genotyping

Four microsatellites markers were used to genotype tapeworms and cysts: SSR09, SSR27, SSR28, and SSR32. Results are shown in Tables 4 and 5, and S1. Because SSR32 was found to be monomorphic, we excluded it from the analysis (S1 Table). Based on sequencing results, we defined that two genotypes were the same if the size bands in the three loci for SSR09, SSR27, and SSR28 were identical. Accordingly, six pigs (P1-P6) had cysts with a genotype identical to tapeworm T3. Two pigs (P7 and P8) had cysts with genotypes not found in any of the tapeworms; both of them were from the same household (#40) (Table 3).

**Table 4:** Genotype of tapeworms found in Pampa Elera community.

Tapeworm	House	Genotype		
		SSR09	SSR27	SSR28
T1	16	160	153	215
T2	69	160	156	218
T3	83	157	153	215
T4	94	160	153	218

Band size determined by sequencing.

**Table 5:** Genotypes of cysts excised from naturally infected pigs based on sequencing.

Pig		Genotype			
	Number of examined cysts	SSR09	SSR27	SSR28	Genotype proportion
P1	13	157	153	215	1.00
	1	^a^	153	215	^b^
	1	^a^	^a^	215	^b^
P2	12	157	153	215	1.00
	1	^a^	153	215	^b^
P3	11	157	153	215	1.00
	1	157	153	^a^	^b^
P4	9	160	153	221	0.90
	1	157	153	215	0.10
P5	8	157	153	215	1.00
	2	^a^	^a^	215	^b^
P6	6	160	153	221	0.60
	4	157	153	215	0.40
P7	9	160	156	215	0.82
	2	160	156	221	0.18
	1	160	^a^	215	^b^
P8	8	160	156	215	0.57
	6	160	156	221	0.43

^a^ Not enough DNA.

^b^ Not considered for genotype proportion calculation.

Two pigs (P4 and P5), that have cysts with genotype matching the genotype of tapeworm T3, lived 220 m away from that tapeworm. The pig P6 that had cysts that matched with tapeworm T3 is the pig that lives furthest away (590 m) from tapeworm T3, other pigs live within that area.

In the field study, cysts recovered from the same pig showed some genetic variation. Four pigs harbored cysts with a unique genotype; however four pigs harbored cysts with two different genotypes, in proportions that ranged from 10% to 43% (Table 5). Based on the spontaneous variability found in the experimental infection, the likelihood that a pig had two different types of cysts in proportions of about 40%/60% is not likely to occur as a natural spontaneous variation (P<0.0001). Therefore we believe that at least two pigs: P8 and P6 harbored cysts with different parental origin (i.e. multiple infections). Hence, 32% of cysts recovered from pig P6 and 35% of cysts recovered from pig P8 are likely to be of different parental origin. The expected heterozygosity ranged from 0 to 0.34 (S2 Table).

### Dendrogram analysis

**Fig 2:**
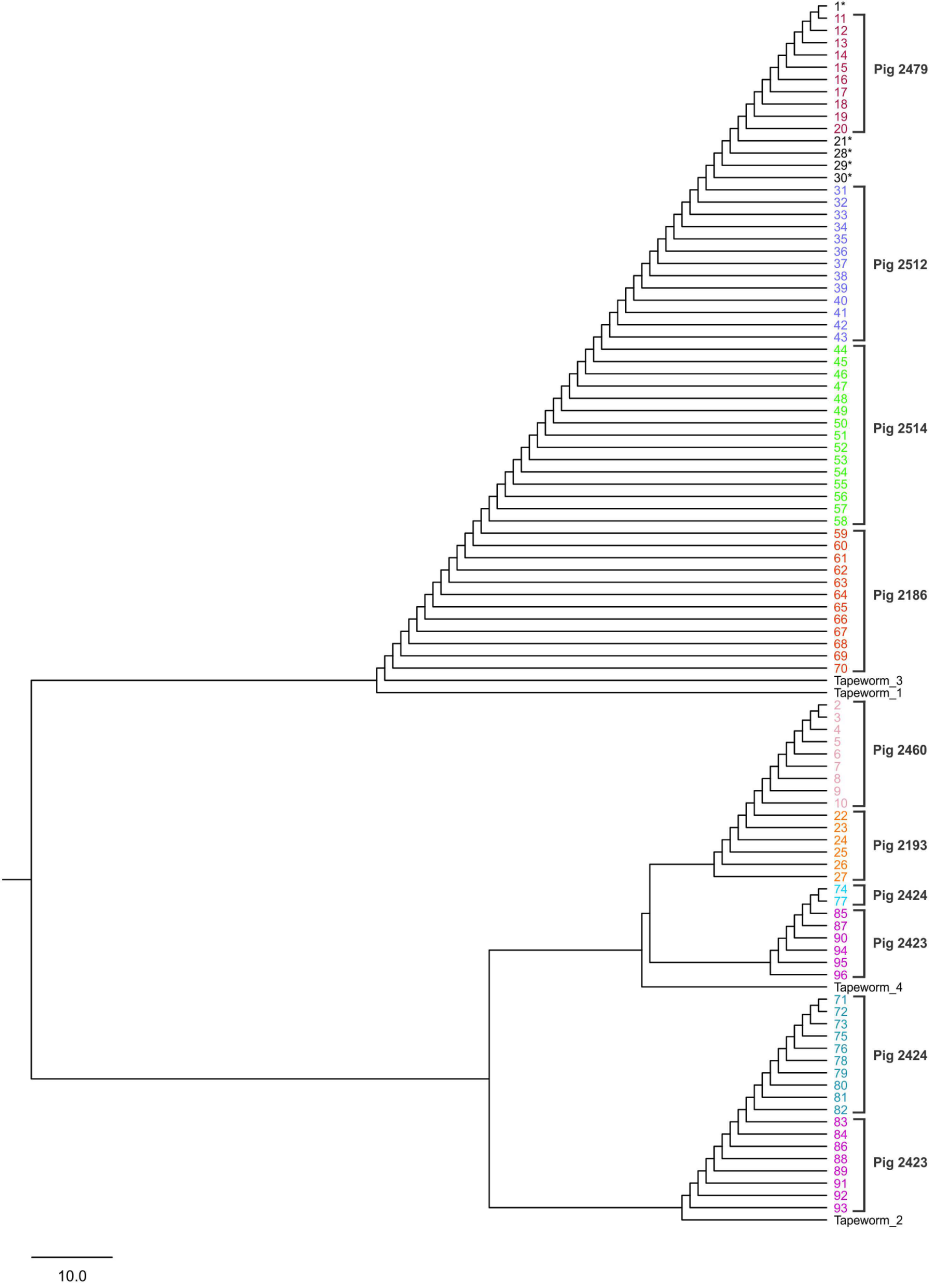
UPGMA dendrogram depicting Dc genetic distances between 96 cysts based on three polymorphic nuclear microsatellite loci. 1–10 (Pig P4), 11–20 (Pig P5), 21–30 (Pig P6), 31–43 (Pig P2), 44–58 (Pig P1), 59–70 (Pig P3), 71–82 (Pig P7), 83–96 (Pig P8). Two main groups are shown.

The UPGMA tree (based on distances computed as differences of the number of “repeated short sequences”), of 8 groups of cysts recovered from different pigs, and four tapeworms based on genetic distances were classified into two groups associated with two regions of different altitude, suggesting that the genetic diversity was related to the geographical location (Fig 2). One group is comprised of the cysts obtained from pigs (P1, P2, P3, and P5) and tapeworm T1 and T3. The second group includes cysts from pigs (P4, P6, and P8), and tapeworms T2 and T4.

To analyze the association of genetic variability and geographical distance, the Mantel test was used for the microsatellite markers. No significant correlation between genetic and geographic distances was found (Spearman Rank correlation coefficient r = 0.166, Mantel P = 0.46).

## Discussion

In this study we show important evidence of genetic variability of *T*. *solium*, and present a promising genotyping tool based on DNA microsatellite markers. First, our data shows that the probability that a cyst has the same genotype (i.e. same microsatellite marker sequence) as the parental tapeworm in an experimental infection is 92.3%. Second, naturally infected pigs in an endemic rural community harboring subtypes of cysts with different genotypes, could be explained by multiple infections. This tool is likely to correctly identify the progeny of a tapeworm.

Defining a genotype as a unique combination of the microsatellite markers, the experimental infection study showed that the progeny of a given tapeworm had an almost identical genotype as the parental worm. This was specially noted in the cysts originating from the tapeworm TB from Cajamarca, in the north of Peru, where all sequenced cysts had exactly the same genotype. This suggests a low genetic variability per generation that can be explained by *T*. *solium* being a monoecious parasite that self-fertilizes [23, 37]. Through our methodology, we can be assured that the cysts (3/39) that displayed different genotypes from the parental tapeworm are due to spontaneous mutations of the microsatellites (e.g. slippage) [38, 39] and not due to experimental PCR, since markers that resulted in different band size by sequencing were genotyped twice, including the PCR reaction itself. The spontaneous mutations in cysts originating from the same tapeworm occurred at a rate of 7.7%, varying less than 2 repeats per generation. Not considering this natural spontaneous genetic variability of cysts, could cause overestimation of multiple infections in pigs; however, higher proportions of different genotypes may point to multiple infections. Despite this natural variation, we show lower genetic variability of *T*. *solium* than previously reported, using RAPD test [14] in an experimental infection. This discrepancy could be explained by the fact that the RAPD test often yields higher variability due to its random nature opposed to a DNA sequencing approach.

The epidemiologic study confirmed genetic variability among cysts obtained from pigs naturally infected within the community, as previously reported [11, 12]. One aim of this study was to show evidence of transmission between tapeworms and cysts using microsatellite markers; important information was obtained from the analysis of the genotypes of cysts obtained from 8 pigs and 4 tapeworms in the community, as explained below.

Two pigs (P7 and P8) from the community had cysts with two microsatellite genotypes different from the identified tapeworms. Since all sacrificed pigs were reported to be born and raised in Pampa Elera, it is likely that they became infected from unidentified tapeworm carriers. Also 23% of people surveyed came from different communities in Piura, Tumbes, and some from Lima. Therefore, it is possible that infections from transient tapeworm carriers may have occurred as well.

Six pigs harbored cysts that matched the genotype of tapeworm T3 (P1-P6), showing some degree of concordance between the tapeworm and cysts recovered from pigs (also shown in the dendrogram); however, the Mantel test did not show a significant association between genetic distances and geography. Nevertheless, it has been shown that pigs can roam several kilometers away from their homes [40, 41]. At this point, it is possible to state that: two or more tapeworms in the community could have the same genotype, and/or the low number of microsatellite markers used does not allow for capture of a finer overall genotype. Genotypes of Tapeworms T1 and T2 did not appear among the excised cysts, the corresponding tapeworm carriers declared having and using a latrine for defecation.

Four pigs harbored one type of cyst and four other pigs harbored two types of cysts. The fact that cysts of one genotype were found within an individual pig may possibly be explained by acquired or protective immunity. The finding of cysts with two genotypes in the proportions found in this study suggest either multiple infections (animals that have eaten food contaminated with two different tapeworms either at the same time or at different times, i.e. reinfection), that the tapeworm had an intrinsic source of variability such as recombination (for instance due to cross fertilization), or that a tapeworm carrier was infected with more than one tapeworm.

Pigs with only one type of cyst were all male and 7 to 9 months old. On the other hand, all pigs that harbored two types of cysts were female, in whom there could be a drop in immunity peri-partum [42], which could favor reinfection. Also, De Aluja *et*.*al*. (1999) reported that experimentally infected pigs were resistant to reinfection for at least five months [43], after which they could become re-infected. All pigs that harbored two types of cysts were older than 9 months old, possibly allowing enough time to be infected twice. Also, it has been reported that pigs from 7 to 9 months old roam greater distances from the household [41], therefore the chances of becoming infected from other sources further from the pigs´ households could be higher. Finally, it was found that two pigs (P7 and P8) that live in the same household had the same two types of cysts. It has been reported by Pray *et*. *al*. (2016) that pigs from the same household have identical roaming ranges and stay together as a herd, possibly explaining why the genotypes were found to be the same.

It is likely that we missed infected pigs and tapeworm carriers. In our previous study [31], in which tapeworm carriers were identified, we invited people from the community and not all were willing to participate. Also, we may have missed tapeworm carriers due to use of an imperfect diagnostic tool (spontaneous sedimentation in tube technique and microscopy) [31]. Regarding pigs, only tongue positive pigs were analyzed, thus it is possible that more infected pigs harboring different cysts are present in the community.

In this study, we initially tested four previously reported polymorphic microsatellite markers; however, based on sequencing analysis, one of them, TS_SSR32, was found to be monomorphic. This finding reinforces conceptions about the lack of precision in the automated capillary system to identify size polymorphisms, as also reported by Manrique et al. [44]. Therefore, we evaluated the genotype of cysts using three microsatellite markers. This number may seem small; however, another study found that a single multilocus microsatellite (the EmtB) of *Echinococcus multilocularis*, was informative enough to genotype strains with a high enough resolution to track transmission [19]. Therefore, it is likely that the three selected microsatellites are sufficient. However further studies are required to evaluate this in a larger population and to include additional microsatellite markers to improve precision to establish relatedness among individuals within a community.

In conclusion, this study shows that these microsatellite markers constitute a promising tool, and further genotyping should be done using additional markers to explore their potential in tracing transmission in an endemic community.

## Supporting information

S1 Table Genotypes of evaluated cysts based on sequencing. (DOCX)

S2 Table Expected heterozygosity of microsatellite markers by group of cysts genotyped from each pig in the rural community. (DOCX)
