# Supplementary material for: Genetic variability of Taenia solium cysticerci recovered from experimentally infected pigs and from naturally infected pigs using microsatellite markers
Source: PLoS Negl Trop Dis. 2017 Dec 28;11(12):e0006087. doi: 10.1371/journal.pntd.0006087 (PMC5746202; doi:10.1371/journal.pntd.0006087)
Supplement: S1 Table — (DOCX) [file pntd.0006087.s001.docx]

**S1 Table. Genotypes of evaluated cysts based on sequencing**

| **Pig** | **Cyst** | **SSR09** | **SSR27** | **SSR28** | **SSR32** |
| --- | --- | --- | --- | --- | --- |
| A1 | 1 | 169 | 168 | 224 | 176 |
|  | 2 | 169 | 168 | 224 | 176 |
|  | 3 | 169 | 168 | 224 | 176 |
|  | 4 | 169 | 168 | 218 | ^a^ |
|  | 5 | 169 | 168 | 224 | 176 |
|  | 6 | 169 | 168 | 224 | ^a^ |
|  | 7 | 169 | 168 | 224 | ^a^ |
|  | 8 | 169 | 168 | 224 | ^a^ |
|  | 9 | 169 | 168 | 224 | ^a^ |
|  | 10 | 169 | 168 | 224 | ^a^ |
|  | 11 | 169 | 165 | 224 | 176 |
|  | 12 | 169 | 168 | 224 | ^b^ |
| A3 | 13 | 169 | 168 | 224 | 176 |
|  | 14 | 169 | 168 | 224 | 176 |
|  | 15 | 169 | 168 | 224 | ^a^ |
|  | 16 | 169 | 168 | 224 | ^a^ |
|  | 17 | 163 | 168 | 224 | ^a^ |
|  | 18 | 169 | 168 | 224 | ^a^ |
|  | 19 | 169 | 168 | 224 | ^b^ |
|  | 20 | 169 | 168 | 224 | ^b^ |
|  | 21 | 169 | 168 | 224 | ^a^ |
|  | 22 | 169 | 168 | 224 | ^a^ |
|  | 23 | 169 | 168 | 224 | ^b^ |
|  | 24 | 169 | 168 | 224 | ^a^ |
|  | 25 | 169 | 168 | 224 | ^b^ |
|  | 26 | 169 | 168 | 224 | ^b^ |
| A7 | 27 | 169 | 168 | 224 | ^a^ |
|  | 28 | 169 | 168 | 224 | ^b^ |
|  | 29 | 169 | 168 | 224 | ^a^ |
|  | 30 | 169 | 168 | 224 | 176 |
|  | 31 | 169 | 168 | 224 | 176 |
|  | 32 | 169 | 168 | 224 | ^a^ |
|  | 33 | 169 | 168 | 224 | ^a^ |
|  | 34 | 169 | 168 | 224 | ^a^ |
|  | 35 | 169 | 168 | 224 | ^a^ |
|  | 36 | 169 | 168 | 224 | ^b^ |
|  | 36 | 169 | 168 | 224 | ^b^ |
|  | 38 | 169 | 168 | 224 | ^b^ |
|  | 39 | 169 | 168 | 224 | ^a^ |
|  | 40 | 169 | 168 | 224 | ^a^ |
| **Pig** | **Cyst** | **SSR09** | **SSR27** | **SSR28** | **SSR32** |
| B4 | 41 | 160 | ^a^ | 221 | 176 |
|  | 42 | 160 | ^a^ | 221 | 176 |
|  | 43 | 160 | 153 | 221 | ^a^ |
|  | 44 | 160 | ^a^ | 221 | ^a^ |
|  | 45 | 160 | 153 | 221 | 176 |
|  | 46 | 160 | 153 | 221 | 176 |
|  | 47 | 160 | 153 | 221 | ^a^ |
|  | 48 | 160 | 153 | 221 | ^b^ |
|  | 49 | 160 | 153 | 221 | ^b^ |
|  | 50 | 160 | 153 | 221 | ^a^ |
|  | 51 | 160 | 153 | 221 | 176 |
| B5 | 52 | 160 | ^a^ | 221 | 176 |
|  | 53 | 160 | 153 | 221 | ^b^ |
|  | 54 | 160 | 153 | 221 | ^a^ |
|  | 55 | 160 | 153 | 221 | 176 |
|  | 56 | 160 | ^a^ | 221 | ^b^ |
| B6 | 57 | 160 | 153 | 221 | ^b^ |
|  | 58 | 160 | 153 | 221 | 176 |
|  | 59 | 160 | 153 | 221 | ^a^ |
|  | 60 | 160 | 153 | 221 | ^b^ |
|  | 61 | 160 | 153 | 221 | ^b^ |
|  | 62 | 160 | 153 | 221 | ^b^ |
|  | 63 | 160 | 153 | 221 | ^b^ |
|  | 64 | 160 | 153 | 221 | ^a^ |
|  | 65 | 160 | 153 | 221 | ^a^ |
|  | 66 | 160 | 153 | 221 | ^a^ |
|  | 67 | 160 | 153 | 221 | ^a^ |
| P1 | 68 | 157 | 153 | 215 | 176 |
|  | 69 | ^a^ | 153 | 215 | 176 |
|  | 70 | ^a^ | ^a^ | 215 | ^a^ |
|  | 71 | 157 | 153 | 215 | 176 |
|  | 72 | 157 | 153 | 215 | 176 |
|  | 73 | 157 | 153 | 215 | 176 |
|  | 74 | 157 | 153 | 215 | ^a^ |
|  | 75 | 157 | 153 | 215 | 176 |
|  | 76 | 157 | 153 | 215 | ^a^ |
|  | 77 | 157 | 153 | 215 | ^b^ |
|  | 78 | 157 | 153 | 215 | ^b^ |
|  | 79 | 157 | 153 | 215 | ^b^ |
|  | 80 | 157 | 153 | 215 | ^a^ |
|  | 81 | 157 | 153 | 215 | ^a^ |
|  | 82 | 157 | 153 | 215 | ^a^ |

| **Pig** | **Cyst** | **SSR09** | **SSR27** | **SSR28** | **SSR32** |
| --- | --- | --- | --- | --- | --- |
| P2 | 83 | 157 | 153 | 215 | 176 |
|  | 84 | 157 | 153 | 215 | 176 |
|  | 85 | 157 | 153 | 215 | 176 |
|  | 86 | 157 | 153 | 215 | 176 |
|  | 87 | 157 | 153 | 215 | 176 |
|  | 88 | 157 | 153 | 215 | 176 |
|  | 89 | 157 | 153 | 215 | ^a^ |
|  | 90 | 157 | 153 | 215 | 176 |
|  | 91 | 157 | 153 | 215 | 176 |
|  | 92 | 157 | 153 | 215 | 176 |
|  | 93 | 157 | 153 | 215 | 176 |
|  | 94 | 157 | 153 | 215 | 176 |
|  | 95 | ^a^ | 153 | 215 | 176 |
| P3 | 96 | 157 | 153 | 215 | 176 |
|  | 97 | 157 | 153 | 215 | 176 |
|  | 98 | 157 | 153 | 215 | 176 |
|  | 99 | 157 | 153 | 215 | 176 |
|  | 100 | 157 | 153 | 215 | 176 |
|  | 101 | 157 | 153 | 215 | 176 |
|  | 102 | 157 | 153 | 215 | 176 |
|  | 103 | 157 | 153 | ^a^ | ^a^ |
|  | 104 | 157 | 153 | 215 | 176 |
|  | 105 | 157 | 153 | 215 | 176 |
|  | 106 | 157 | 153 | 215 | 176 |
|  | 107 | 157 | 153 | 215 | 176 |
| P4 | 108 | 157 | 153 | 215 | 176 |
|  | 109 | 160 | 153 | 221 | 176 |
|  | 110 | 160 | 153 | 221 | 176 |
|  | 111 | 160 | 153 | 221 | 176 |
|  | 112 | 160 | 153 | 221 | 176 |
|  | 113 | 160 | 153 | 221 | 176 |
|  | 114 | 160 | 153 | 221 | 176 |
|  | 115 | 160 | 153 | 221 | 176 |
|  | 116 | 160 | 153 | 221 | 176 |
|  | 117 | 160 | 153 | 221 | 176 |
| P5 | 118 | 157 | 153 | 215 | 176 |
|  | 119 | 157 | 153 | 215 | 176 |
|  | 120 | 157 | 153 | 215 | 176 |
|  | 121 | 157 | 153 | 215 | 176 |
|  | 122 | 157 | 153 | 215 | 176 |
|  | 123 | 157 | 153 | 215 | 176 |
|  | 124 | 157 | 153 | 215 | 176 |
|  | 125 | ^a^ | ^a^ | 215 | 176 |
| **Pig** | **Cyst** | **SSR09** | **SSR27** | **SSR28** | **SSR32** |
| P5 | 126 | ^a^ | ^a^ | 215 | 176 |
|  | 127 | 157 | 153 | 215 | 176 |
| P6 | 128 | 157 | 153 | 215 | 176 |
|  | 129 | 160 | 153 | 221 | 176 |
|  | 130 | 160 | 153 | 221 | 176 |
|  | 131 | 160 | 153 | 221 | 176 |
|  | 132 | 160 | 153 | 221 | 176 |
|  | 133 | 160 | 153 | 221 | 176 |
|  | 134 | 160 | 153 | 221 | 176 |
|  | 135 | 157 | 153 | 215 | 176 |
|  | 136 | 157 | 153 | 215 | 176 |
|  | 137 | 157 | 153 | 215 | 176 |
| P7 | 138 | 160 | 156 | 215 | 176 |
|  | 139 | 160 | ^a^ | 215 | 176 |
|  | 140 | 160 | 156 | 215 | 176 |
|  | 141 | 160 | 156 | 221 | 176 |
|  | 142 | 160 | 156 | 215 | 176 |
|  | 143 | 160 | 156 | 215 | 176 |
|  | 144 | 160 | 156 | 221 | 176 |
|  | 145 | 160 | 156 | 215 | 176 |
|  | 146 | 160 | 156 | 215 | 176 |
|  | 147 | 160 | 156 | 215 | 176 |
|  | 148 | 160 | 156 | 215 | 176 |
|  | 149 | 160 | 156 | 215 | 176 |
| P8 | 150 | 160 | 156 | 215 | 176 |
|  | 151 | 160 | 156 | 215 | 176 |
|  | 152 | 160 | 156 | 221 | 176 |
|  | 153 | 160 | 156 | 215 | 176 |
|  | 154 | 160 | 156 | 221 | 176 |
|  | 155 | 160 | 156 | 215 | 176 |
|  | 156 | 160 | 156 | 215 | 176 |
|  | 157 | 160 | 156 | 221 | 176 |
|  | 158 | 160 | 156 | 215 | 176 |
|  | 159 | 160 | 156 | 215 | 176 |
|  | 160 | 160 | 156 | 215 | 176 |
|  | 161 | 160 | 156 | 221 | 176 |
|  | 162 | 160 | 156 | 221 | 176 |
|  | 163 | 160 | 156 | 221 | 176 |

Cysts are grouped by pig from which they were extracted. Experimentally infected pigs named A1, A3, or A7 were infected by proglottids from tapeworm TA, and pigs named B4, B5, and B6 were infected by proglottids from Tapeworm TB. Naturally infected pigs are coded by P1, P2, P3, P4, P5, P6, P7, and P8.

^a^ Not enough DNA.  ^b^ Not evaluated.
