# Supplementary material for: Genetic variability of Taenia solium cysticerci recovered from experimentally infected pigs and from naturally infected pigs using microsatellite markers
Source: PLoS Negl Trop Dis. 2017 Dec 28;11(12):e0006087. doi: 10.1371/journal.pntd.0006087 (PMC5746202; doi:10.1371/journal.pntd.0006087)
Supplement: S2 Table — (DOCX) [file pntd.0006087.s002.docx]

**S2 Table. Expected heterozygosity of microsatellite markers by group of cysts genotyped from each pig in the rural community**

| Locus# | P1 | P2 | P3 | P4 | P5 | P6 | P7 | P8 | Mean | s.d. |
| --- | --- | --- | --- | --- | --- | --- | --- | --- | --- | --- |
| SSR09 | 0.00000 | 0.00000 | 0.00000 | 0.18947 | 0.00000 | 0.50526 | 0.00000 | 0.00000 | 0.05789 | 0.15105 |
| SSR27 | 0.00000 | 0.00000 | 0.00000 | 0.00000 | 0.00000 | 0.00000 | 0.00000 | 0.00000 | 0.00000 | 0.00000 |
| SSR28 | 0.00000 | 0.00000 | 0.00000 | 0.18947 | 0.00000 | 0.50526 | 0.28986 | 0.50794 | 0.12438 | 0.20171 |
| Mean | 0.00000 | 0.00000 | 0.00000 | 0.12632 | 0.00000 | 0.33684 | 0.09662 | 0.16931 | 0.06076 | 0.10580 |
| s.d. | 0.00000 | 0.00000 | 0.00000 | 0.10939 | 0.00000 | 0.29171 | 0.16735 | 0.29326 | 0.07181 | 0.11646 |

Cysts are grouped by pig from which were extracted. Pigs are named as P1 – P8
